# Contributions to the ‘noise floor’ in gene expression in a population of dividing cells

**DOI:** 10.1038/s41598-020-69217-2

**Published:** 2020-08-11

**Authors:** Jakub Jędrak, Anna Ochab-Marcinek

**Affiliations:** Institute of Physical Chemistry, Polish Academy of Sciences, ul. Kasprzaka 44/52, 01-224 Warsaw, Poland

**Keywords:** Biophysics, Biological physics

## Abstract

Experiments with cells reveal the existence of a lower bound for protein noise, the noise floor, in highly expressed genes. Its origins are still debated. We propose a minimal model of gene expression in a proliferating bacterial cell population. The model predicts the existence of a noise floor and it semi-quantitatively reproduces the curved shape of the experimental noise vs. mean protein concentration plots. When the cell volume increases in a different manner than does the mean protein copy number, the noise floor level is determined by the cell population’s age structure and by the dependence of the mean protein concentration on cell age. Additionally, the noise floor level may depend on a biological limit for the mean number of bursts in the cell cycle. In that case, the noise floor level depends on the burst size distribution width but it is insensitive to the mean burst size. Our model quantifies the contributions of each of these mechanisms to gene expression noise.

## Introduction

Experimental data for bacteria^1–3^ and yeast^4–7^ show that, for proteins of low abundance, the coefficient of variation (variance divided by mean squared) of protein molecule copy number or concentration is a decreasing function of average protein copy number or concentration. However, for highly expressed genes, the coefficient of variation tends to a constant level^1–8^. This lower bound for protein noise is called *noise floor*. There is a debate in the literature over the origin of the noise floor^9^: It has been attributed to protein partitioning at cell division^7^ or to the existence of some limits to the frequencies of transcriptional and translational bursting^10^. In Ref.^1^, the noise floor was introduced by heuristic addition of extrinsic noise as an overlay to the existing model.

Here, we introduce a model of gene expression combining the effects which, to date, have been studied separately: cell volume growth^11,12^, protein partitioning at cell division^12–16^, age structure of the cell population^11–13,15,16^, and dependence of protein production on cell age^17,18^. These ingredients suffice to semi-quantitatively reproduce the ’boomerang’ shape of the experimental plots of noise vs protein concentration.

We show that, in a proliferating cell population, the noise floor is always present if the mean protein concentration in cells depends on their cell cycle age $\tau$, i.e., if the mean protein copy number increases in a different manner than the cell volume. This can be the case even if the transcription rate *k* is constant during the cell cycle: The mean protein copy number increases linearly in time but the cell volume may increase, e.g., exponentially. We also show that the dependence of *k* on $\tau$ can considerably increase the noise floor level.

## Model

**Table 1:** Notation.

Probability density function	Describes the distribution of	Variable	Moments or averages	Eqs.
*p*(*x*, *t*)	Protein copy number *x* in a cell lineage at time *t*	$x \in [0, \infty )$	$\mu _r(t)\equiv \int _0^{\infty } x^r p(x,t)dx$	
$p(x|\tau )$	Protein copy number *x* in a single cell of age $\tau$	$x \in [0, \infty )$	$\mu _r(\tau )\equiv \int _0^{\infty } x^r p(x|\tau )dx$	(13)
$\nu (u, t)$	Protein burst size *u* in a cell lineage at time *t*	$u \in [0, \infty )$	$m_r(t)\equiv \int _0^{\infty } u^r \nu (u,t)dx$	
$\nu (u|\tau )$	Protein burst size *u* in a single cell of age $\tau$	$u \in [0, \infty )$	$m_r(\tau )= \int _{0}^{\infty } u^r \nu (u|\tau ) du$	(9)
$\eta (q)$	Protein partitioning ratio *q*	$q \in [0, 1]$	${M}_r \equiv \int _0^1 q^r\eta (q)dq$	(12)
$\phi _a(\tau )$	Cell age $\tau$	$\tau \in [0, T]$	$\langle f(\tau ) \rangle _a \equiv \int _{0}^{T} f(\tau )\phi _a(\tau ) d\tau$	(21)
$p_a(x) \equiv \langle p(x|\tau ) \rangle _a$	Protein copy number *x* in cell population	$x \in [0, \infty )$	$\mu _{ra} \equiv \int _0^{\infty } x^r p_a(x)dx$	
${\tilde{p}}({\tilde{x}}|\tau )$	Protein concentration ${\tilde{x}}$ in a single cell of age $\tau$	${\tilde{x}} \in [0, \infty )$	${\tilde{\mu }}_r(\tau ) = \int _{0}^{\infty } {\tilde{x}}^r {\tilde{p}}({\tilde{x}}|\tau )d{\tilde{x}}$	(26)
${\tilde{p}}_a({\tilde{x}}) \equiv \langle {\tilde{p}}({\tilde{x}}|\tau ) \rangle _a$	Protein concentration ${\tilde{x}}$ in cell population	${\tilde{x}} \in [0, \infty )$	${\tilde{\mu }}_{ra} \equiv \int _0^{\infty } {\tilde{x}}^r {\tilde{p}}_a({\tilde{x}})d{\tilde{x}}$	(30)
$p^{\star }_a (x^{\star })$	Effective protein copy number $x^{\star }$ in cell population	$x^{\star } \in [0, \infty )$	$\mu ^{\star }_{ar} = \int _{0}^{\infty } (x^{\star })^{r} p^{\star }_a (x^{\star })d x^{\star }$	(36)

The model is based on a Master equation which describes the time evolution of the probability that there are *x* protein molecules present in a single cell at time *t* between cell divisions. This probability is given by the probability density function *p*(*x*, *t*). We assume that the protein copy number randomly increases due to translational bursts and it randomly decreases due to cell division. The random time intervals between bursts are drawn from a distribution dependent on the transcription rate *k*(*t*). Each burst has a random size *u* drawn from a distribution $\nu (u,t)$. Even though we are unable to obtain the analytical solution for the probability density function *p*(*x*, *t*), we can get cumulants and moments of *p*(*x*, *t*) from its Laplace transform.

Next, we consider the effect of random protein partitioning at cell division, which is described by the probability density function $\eta (q)$: *q* is a fraction of protein molecules inherited by one of the daughter cells. We assume that cell divisions are instantaneous and occur periodically (cell cycle duration *T* is always the same). These ingredients are sufficient to describe a single cell lineage. In order to describe the whole proliferating population we average the quantities referring to a single cell line with the cell age distribution (the population age structure). To be able to compare our model with the experimental results of Ref.^1^, we have to change the variables from protein copy number to protein concentration and, subsequently, from protein concentration to the effective protein copy number, being equal to the protein concentration multiplied by the average cell volume in cell population. For the growing and dividing cell that belongs to a proliferating cell population, the relationship between protein copy number and protein concentration is no longer trivial, and one must carefully distinguish between the corresponding quantities (see Table 1 for probability density functions and their moments).

### Protein production

Consider a single cell lineage with cell divisions at $t=t_1, t_2, \ldots , t_n$. *p*(*x*, *t*)*dx* is the probability that there are *x* protein molecules in the cell at time *t*. Here, $x \in [0, \infty )$ is a continuous approximation of the discrete protein copy number and not protein concentration. Between cell division events, time evolution of *p*(*x*, *t*) is given by the chemical master equation^19–21^

1 $$\begin{aligned} \partial _t p(x,t) = k(t) \int _{0}^{x} w(x-x^{\prime }, t) p(x^{\prime },t) dx^{\prime }. \end{aligned}$$

Equation (1) describes a stochastic protein production in random bursts; *k*(*t*) is the transcription rate, $u=x-x^{\prime }$ is the burst size, $w(u, t) \equiv \nu (u, t) - \delta (u)$, $\nu (u,t)$ is the burst size probability density function, and $\delta (u)$ is Dirac delta^19–21^. We neglect protein degradation, because in bacteria like *E. coli* most proteins are stable. The Laplace transform (${\mathscr {L}}\{\ldots \}$) of Eq. (1) reads

2 $$\begin{aligned} \partial _t G(s, t) = k(t){\hat{w}}(s, t) G(s, t), \end{aligned}$$

where $G(s, t)={\mathscr {L}}[p(x, t)]\equiv \int _0^{\infty } e^{-s x} p(x,t) d x$, ${\hat{w}}(s, t)={\hat{\nu }}(s, t)-1$ and ${\hat{\nu }}(s, t)={\mathscr {L}}\{\nu (u, t)\}\equiv \int _0^{\infty } e^{-s u} \nu (u, t) d u$; note that $\ln [G(-s,t)] = \sum _{r=1}^{\infty } \kappa _r(t) s^r/r!$ is a cumulant generating function for *p*(*x*, *t*). Equation (2) will be used to obtain the time-evolution of cumulants $\kappa _r(t)$ of *p*(*x*, *t*) during the cell cycle. Equation (2) can be easily solved, one gets

3 $$\begin{aligned} G(s, t)= G(s, t_0) \exp \left( \int _{t_0}^{t}k(t^{\prime }){\hat{w}}(s, t^{\prime }) dt^{\prime } \right) , \end{aligned}$$

where $G(s, t_0)= {\mathscr {L}}[p(x, t_0)]$. However, in most cases it is not possible to compute $p(x, t) = {\mathscr {L}}^{-1}[G(s, t)]$ analytically.

### Protein partitioning at cell division

At cell division (assumed to be instantaneous), the time evolution of *p*(*x*, *t*) given by Eqs. (1) or (2) is interrupted and protein molecules are partitioned between daughter cells: $x\rightarrow \{qx, (1-q)x\}$. Here, $0 \le q \le 1$ is a random number, drawn from the probability density function $\eta (q)=\eta (1-q)$. Protein partitioning implies the following relations^22^:

4 $$\begin{aligned} \lim _{\delta _t \rightarrow 0} p(x, \,t_i + \delta _t)= & {} \lim _{\delta _t \rightarrow 0} \int _{0}^{1} \frac{\eta (q)}{q} p\left( \frac{x}{q},\, t_i - \delta _t \right) dq, \end{aligned}$$

5 $$\begin{aligned} \lim _{\delta _t \rightarrow 0} G(s, \,t_i + \delta _t)= & {} \lim _{\delta _t \rightarrow 0} \int _{0}^{1} \eta (q) G(qs, \, t_i - \delta _t) dq. \end{aligned}$$

Equation (5) links the Laplace transforms of the protein copy number probability density functions immediately after and before cell division, during which protein molecules are randomly partitioned between daughter cells.

From now on, we assume that cell division occurs periodically every time *T*^12,13,15^ and we denote by $\tau$ the age of a cell within a single cell cycle. We treat both *k*(*t*) and ${\hat{w}}(s, t)$ as periodic functions of observation time *t*; $k(t) = k(t+T)$, ${\hat{w}}(s, t) = {\hat{w}}(s, t+T)$ and assume that the number of cell divisions is large enough to neglect the influence of initial conditions at $t=0$. In consequence, $p(x, t)= p(x, t+T)$, $G(s, t)=G(s, t+T)$. Now we consider $t \in (t_i, t_i + T)$ for some *i* and *p*(*x*, *t*) is interpreted as a conditional probability density function of finding *x* protein molecules in cell at the age $\tau =t-t_i \in (0, T)$. To stress this, we change the notation:

6 $$\begin{aligned} t \rightarrow \tau , \,\,\, p(x, t) \rightarrow p(x|\tau ), \,\,\, G(s, t) \rightarrow G(s|\tau ), \,\,\, \nu (u, t) \rightarrow \nu (u|\tau ), \,\,\, k(t) \rightarrow k(\tau ), \end{aligned}$$

and analogously for other functions of *t*, which are now treated as functions of $\tau$. For any function $f(\tau )$ we define the notation of its values just after and just before cell division: $f(0) \equiv \lim _{\tau \rightarrow 0^{+}} f(\tau )$ and $f(T) \equiv \lim _{\tau \rightarrow T^{-}} f(\tau )$.

### Cumulants of protein copy number distribution depend on moments of burst size distribution

In this subsection, we show that the increase in the *r*-th cumulant $\kappa _r(\tau )$ of the protein copy number probability density function $p(x|\tau )$ during the cell cycle depends solely on the time evolution of the *r*-th moment $m_r(\tau )$ of the burst size probability density function $\nu (u|\tau )$. From (6) it follows that if cell age $\tau$ and not the observation time *t* is used as the time variable, Eq. (2) reads

7 $$\begin{aligned} \partial _{\tau } G(s|\tau ) = k(\tau ){\hat{w}}(s|\tau ) G(s|\tau ). \end{aligned}$$

By dividing Eq. (7) by $G(s|\tau )$ one gets the time-evolution equation for $\ln [G(s|\tau )]$ and hence the time-evolution equation obeyed by cumulants $\kappa _r(\tau )$ of $p(x|\tau )$ (note that we have changed the interpretation of time variable $t \rightarrow \tau$ and therefore the notation according to Eq. (6)):

8 $$\begin{aligned} \frac{d {\kappa }_r(\tau )}{d \tau } - k(\tau )m_r(\tau ) = 0, \end{aligned}$$

where

9 $$\begin{aligned} m_r(\tau )= \int _{0}^{\infty } u^r \nu (u|\tau ) du \end{aligned}$$

is the *r*-th moment of the burst size probability density function $\nu (u|\tau )$. From Eq. (8) it follows that

10 $$\begin{aligned} \kappa _r(\tau )-\kappa _r(0) = \int _{0}^{\tau }k(\tau ^{\prime })m_r(\tau ^{\prime })d\tau ^{\prime } \equiv {J}_r(\tau ). \end{aligned}$$

### Moments of protein copy number distribution depend on moments of protein partitioning distribution and burst size distribution

In this subsection, we link the time evolution of $\mu _1(\tau )$ and $\mu _2(\tau )$ (the first and second moments of the protein copy number probability density function $p(x|\tau )$ for a single cell) with the moments of $\nu (u|\tau )$ and $\eta (q)$ (the burst size and protein partitioning probability density functions).

First, we notice the relation between the *r*-th moments of $p(x|\tau )$ immediately after and before cell division with the *r*-th moment of $\eta (q)$:

11 $$\begin{aligned} \mu _r(0) = \mu _r(T) {M}_r, \end{aligned}$$

where

12 $$\begin{aligned} {M}_r \equiv \int _0^1 q^r\eta (q)dq \end{aligned}$$

is the *r*-th moment of the protein partitioning probability density function $\eta (q)$ and

13 $$\begin{aligned} \mu _r(\tau )\equiv \int _0^{\infty } x^r p(x|\tau )dx \end{aligned}$$

is the *r*-th moment of the protein copy number probability distribution $p(x|\tau )$. The relation (11) follows from the Eq. (4) or from the Eq. (5), which links the Laplace transform of $p(x|\tau )$ just after the cell division with the Laplace transform of $p(x|\tau )$ just before the division. Note that $2^{-r} \le {M}_r \le 2^{-1}$ and ${M}_1 = 1/2$ due to the assumed symmetry of $\eta (q)$: $\eta (q)=\eta (1-q)$. (On average, each daughter cell obtains half of the mother’s protein molecules of a given type.)

Now we want to link the moments $\mu _1(\tau )$ and $\mu _2(\tau )$ with $M_2$, $m_1(\tau )$, and $m_2(\tau )$. For this, we use the auxiliary functions,

14 $$\begin{aligned} h_1({M}_2) = \frac{4{M}_2 - 1}{1 - {M}_2}, \,\,\, h_2({M}_2) = \frac{{M}_2}{1-{M}_2}, \end{aligned}$$

as well as Eq. (11) and the definition of ${J}_r(\tau )$, given by the Eq. (10), with

15 $$\begin{aligned} \,{I}_r = {J}_r(T). \end{aligned}$$

The first two moments of $p(x|\tau )$ can be written as:

16 $$\begin{aligned} \mu _1(\tau )= & {} \,{I}_1 + {J}_1(\tau ),\nonumber \\ \mu _2(\tau )= & {} h_2({M}_2){I}_2 + {J}_2(\tau ) + h_1({M}_2){I}^2_1 + \mu ^2_1(\tau ). \end{aligned}$$

Here, $h_1( {M}_2)$ and $h_2( {M}_2)$ depend on the second moment ${M}_2$ of the protein partitioning probability density function $\eta (q)$, whereas $\,{I}_1$, ${J}_1(\tau )$, $\,{I}_2$, ${J}_2(\tau )$ contain the dependence on the first and second moments $m_1(\tau )$, $m_2(\tau )$ of the burst size probability density function $\nu (u|\tau )$. Note that $\mu _1(\tau )$ and $\mu _2(\tau )$ (16) obey the boundary conditions imposed by Eq. (11). In particular, we have

17 $$\begin{aligned} \mu _1(0) = {I}_1, \,\,\,\, \mu _1(T) = 2{I}_1, \end{aligned}$$

as the mean protein copy number doubles during the cell cycle.

The time evolution of $\mu _1(\tau )$ and $\mu _2(\tau )$ for a single cell, as given by Eq. (16), will be needed in the next subsections to obtain the quantities referring to the whole proliferating cell population.

### Emulating binomial protein partitioning distribution

Protein partitioning statistics $\eta (q)$ used here does not depend on *x*(*T*), the number of protein molecules present in the cell immediately before cell division^22–24^. Still, we can choose an arbitrary $\eta (q)$. We use this freedom to impose the following constraint on ${M}_2$:

18 $$\begin{aligned} \mu _2(T) + \mu _1(T) = 4 \mu _2(0), \end{aligned}$$

which holds for the binomial distribution describing protein partitioning in models that assume a discrete protein copy number^12,13,15^. By enforcing the constraint given by Eq. (18) we emulate the behaviour of the first and second moments of the binomial distribution because only this information about the partitioning statistics is needed within our model to calculate the coefficients of variation of protein copy number or concentration.

Using (11), (16), and the constraint (18), we present the second moment ${M}_2$ of the protein partitioning probability density function $\eta (q)$ as dependent on the first two moments $m_1(\tau )$, $m_2(\tau )$ of the burst size probability density function $\nu (u|\tau )$ (through ${I}_1$ and ${I}_2$):

19 $$\begin{aligned} M_2({I}_1, {I}_2)= & {} \frac{{I}_2 + 3{I}^2_1 + 2 {I}_1}{4 {I}_2 + 12 {I}^2_1 + 2 {I}_1}. \end{aligned}$$

Equation (19) is valid as long as $1 \le 4 {M}_2({I}_1, {I}_2) \le 2$.

From (11) and (18) yet another property of the binomial partitioning follows for the coefficient of variation of the protein copy number: $c_v^2(0)-c_v^2(T) = (2 {I}_1)^{-1} = 1/{\mu _1(T)}$^14^, where

20 $$\begin{aligned} c_v^2(\tau )= \frac{\mu _2(\tau ) - \mu _1^2(\tau )}{\mu _1^2(\tau )} = \frac{\kappa _2(\tau )}{\mu _1^2(\tau )}. \end{aligned}$$

The relation (19), linking $M_2$, $m_1(\tau )$, and $m_2(\tau )$, will be later needed for the Eq. (32) which defines ${M}_2$ for a particular choice of the burst size probability density function $\nu (u|\tau )$, which will further serve to derive the coefficient of variation of protein concentration for that case.

### Population averaging over the age structure

All the mathematical results obtained so far referred to a single cell or to a cell lineage. In order to obtain the protein copy number probability density function $p_a(x)$ for the proliferating cell population, we must average $p(x|\tau )$ or its moments $\mu _r(\tau )$ over the cell age probability density function (population age structure) $\phi _a(\tau )$^11–13,15^. We assume here that the environmental conditions are constant, the population has reached the state of balanced growth and its age structure is stationary; $\phi _a(t,\tau )=\phi _a(\tau )$. (The time independence of the population age structure $\phi _a(\tau )$ is neither guaranteed, nor obvious. Also, the convergence of $\phi _a(t,\tau )$ to the stationary age distribution $\phi _a(\tau )$ may be nontrivial^25,26^.)

For any function $f(\tau )$ we introduce the following notation for the population average over the cell age probability density function:

21 $$\begin{aligned} \langle f(\tau ) \rangle _a \equiv \int _{0}^{T} f(\tau )\phi _a(\tau ) d\tau . \end{aligned}$$

The population average $\langle f(\tau ) \rangle _a$ (21) should not be confused with averages over sub-population of cells of the same age or with the time average of $f(\tau )$, $\overline{f(\tau )} \equiv \frac{1}{T}\int _{0}^{T} f(\tau ) d\tau$ over a single cell cycle. Only for the homogeneous age structure, $\phi _a(\tau )= 1/T$, we have $\overline{f(\tau )} = \langle f(\tau ) \rangle _a$.

For an exponentially growing population in the state of balanced growth, $\phi _a(\tau )$ is given by^11–13, 15,27^

22 $$\begin{aligned} \phi _a(\tau ) = \frac{2 \ln 2}{T} \exp \left( - \frac{\ln 2}{T} \tau \right) . \end{aligned}$$

Note that in order to describe the gating procedure—the selection of cells with similar cell age or size—it is sufficient within the present approach to consider an appropriately modified age structure for the whole population: The domain of $\phi _a(\tau )$ given by Eq. (22), i.e., the interval [0, *T*] has to be restricted to some narrower age range, $\tau \in (\tau _A, \tau _B)$ with $0< \tau _A< \tau _B < T$.

The averaging procedure defined by Eq. (21) is not the most general way to obtain the quantities referring to the whole cell population from those referring to a single cell line. A more general approach would involve other model parameters being random variables (e.g., cell volume, cell cycle length), so that the age structure $\phi _a(\tau )$ or the Master equation for protein levels would be derived ’from scratch’ from the time evolution of these variables^28–30^.

### Moments of the protein copy number distribution after integration over the age structure

In order to get the total variance of the protein copy number, i.e., the variance of $p_a(x) \equiv \langle p(x|\tau ) \rangle _a$ referring to the whole cell population, we need the moments of the protein copy number distribution $p(x|\tau )$ to be integrated over the age structure according to Eq. (21): $\mu _{1a} = \kappa _{1a} \equiv \langle \mu _1(\tau ) \rangle _a$ and $\mu _{2a}\equiv \langle \mu _2(\tau )\rangle _a$. We obtain

23 $$\begin{aligned} \kappa _{2a}\equiv & {} \mu _{2a} - \mu ^2_{1a} = \langle {J}_2(\tau ) \rangle _a + h_2( {M}_2) \,{I}_2 + h_1( {M}_2) \,{I}^2_1 + \text {var}_{a}[{\mu }_1(\tau )], \end{aligned}$$

where $h_1({M}_2)$, $h_2({M}_2)$ are given by (14) and ${M}_2$ is given by (19). The last term of (23), $\text {var}_{a}[{\mu }_1(\tau )] = \langle {\mu }^{2}_{1}(\tau ) \rangle _a - \langle {\mu }_1(\tau ) \rangle _a^2$, is the variance of the mean protein copy number $\mu _1(\tau )$ (16) computed with respect to $\phi _a(\tau )$ using (21). This follows from the law of total variance^15^, with $\phi _a(\tau )$ playing the role of a mixing distribution and $p(x|\tau )$ being the conditional distribution. Note that, due to the boundary conditions (17), we have $\text {var}_{a}[{\mu }_1(\tau )] > 0$.

### Protein concentration

Until now, we have been considering cellular protein levels in terms of the molecule copy number *x*. Here, we re-calculate the moments of the protein copy number probability density function $p(x|\tau )$ into the moments of the probability density function ${\tilde{p}}({\tilde{x}}|\tau )$ for protein concentration ${\tilde{x}}$.

The growing and dividing cell changes its volume, which leads to the following relationship between the protein copy number $x(\tau )$ and protein concentration ${\tilde{x}}(\tau )$ during the cell cycle:

24 $$\begin{aligned} {\tilde{x}}(\tau ) \equiv \frac{x(\tau )}{V(\tau )}, \end{aligned}$$

where $V(\tau )$ denotes the volume of a cell of age $\tau \in (0, T)$ and we assume that $V(T) = 2 V(0)$.

We have $p(x|\tau )dx = {\tilde{p}}({\tilde{x}}|\tau )d{\tilde{x}}$, hence the probability density functions for protein copy number and protein concentration scale as

25 $$\begin{aligned} {\tilde{p}}({\tilde{x}}|\tau ) = V(\tau ) p(V(\tau ){\tilde{x}}|\tau ), \end{aligned}$$

and analogously does the concentration burst size probability density function ${\tilde{\nu }}({\tilde{u}}|\tau )$. The relationship between the moments of $p(x|\tau )$ and those of ${\tilde{p}}({\tilde{x}}|\tau )$ reads

26 $$\begin{aligned} {\tilde{\mu }}_r(\tau ) \equiv \int _{0}^{\infty } {\tilde{x}}^r {\tilde{p}}({\tilde{x}}|\tau )d{\tilde{x}} = \frac{\mu _r(\tau )}{[V(\tau )]^{r}}. \end{aligned}$$

Mean protein concentration does not change during cell division,

27 $$\begin{aligned} {\tilde{\mu }}_1(0) = \frac{{\mu }_1(0)}{V(0)} = \frac{{I}_1}{V(0)} = \frac{2{I}_1}{2V(0)} = \frac{{\mu }_1(T)}{V(T)} = {\tilde{\mu }}_1(T), \end{aligned}$$

yet individual cells of the same age $\tau$ differ with respect to the protein concentration ${\tilde{x}}(\tau )$. The variance of the protein concentration ${\tilde{x}}$ computed for the whole proliferating cell population reads

28 $$\begin{aligned} {\tilde{\kappa }}_{2a}\equiv & {} {\tilde{\mu }}_{2a} - {\tilde{\mu }}^2_{1a} = \langle {J}_2(\tau ) [V(\tau )]^{-2} \rangle _a + h_2( {M}_2) \,{I}_2 \langle [V(\tau )]^{-2} \rangle _a + h_1( {M}_2) \,{I}^2_1 \langle [V(\tau )]^{-2} \rangle _a + \text {var}_{a}[{\tilde{\mu }}_1(\tau )]. \end{aligned}$$

In the above, ${\tilde{\mu }}_{1a} = \langle {\tilde{\mu }}_1(\tau ) \rangle _a$ and ${\tilde{\mu }}_{2a} = \langle {\tilde{\mu }}_2(\tau ) \rangle _a$ are the first and second moments of ${\tilde{p}}_a({\tilde{x}})$ defined as

29 $$\begin{aligned} {\tilde{p}}_a({\tilde{x}}) \equiv \langle {\tilde{p}}({\tilde{x}}|\tau ) \rangle _a \equiv \int _{0}^{T} {\tilde{p}}({\tilde{x}}|\tau )\phi _a(\tau ) d\tau , \end{aligned}$$

whereas $\text {var}_{a}[{\tilde{\mu }}_1(\tau )] = \langle {\tilde{\mu }}^2_1({\tau }) \rangle _a - \langle {\tilde{\mu }}_1(\tau ) \rangle _a^2$. Clearly, ${\tilde{\mu }}_{1a}$ and ${\tilde{\mu }}_{2a}$ are also moments of ${\tilde{p}}({\tilde{x}}|\tau )$ (25) averaged with population age structure $\phi _a(\tau )$ using Eq. (21),

30 $$\begin{aligned} {\tilde{\mu }}_{ra} \equiv \int _0^{\infty } {\tilde{x}}^r {\tilde{p}}_a({\tilde{x}})d{\tilde{x}} = \int _0^{\infty } \int _{0}^{T} {\tilde{x}}^r {\tilde{p}}({\tilde{x}}|\tau )\phi _a(\tau ) d\tau d{\tilde{x}} = \int _{0}^{T} {\tilde{\mu }}_r(\tau ) \phi _a(\tau ) d\tau = \langle {\tilde{\mu }}_r(\tau ) \rangle _a, \,\,\,\, r = 1, 2. \end{aligned}$$

Note that Eq. (28) follows from the law of total variance, as in the case of protein copy number and Eq. (23).

For protein concentration, $\text {var}_{a}[{\tilde{\mu }}_1(\tau )] = 0$ if ${\tilde{\mu }}_1(\tau )= {\tilde{\mu }}_{1a}$ (i.e., if the mean protein copy number is proportional to the cell volume). This is in contrast to the case of the protein copy number, where $\text {var}_{a}[{\mu }_1(\tau )] = 0$ is impossible due to the boundary conditions (17).

### Distribution of burst sizes

Translational bursting was directly observed by the Xie group in production of reporter proteins under the control of a repressed *lac* promoter in *E. coli*. The distributions of protein burst sizes were exponential^31–33^. In this subsection, we assume a more general form of the burst size probability density function, which also includes the exponential one: From now on, we consider the $\tau$-independent burst size probability density function of the form

31 $$\begin{aligned} \nu (u)=y\left( u/b \right) /b, \end{aligned}$$

where $b = m_1$ is the mean burst size and $m_2 = C_2 b^2$. (For the exponential probability density function, $y(z)=\exp (-z)$ and $C_2=2$.) Then, the Eq. (19), which links the moments of the probability density functions for protein partitioning and for the burst sizes, reads

32 $$\begin{aligned} {M}_2= & {} \frac{1}{4} + \frac{3}{4\left[ 1 + 2 C_2 b + 3 \mu _1(T)\right] }. \end{aligned}$$

Note that ${M}_2 \le 1/2$ as long as $2 C_2 b + 3 \mu _1(T) \ge 2$, which is the case for all but the very small *b* and $\mu _1(T)$, where the continuous approximation to the discrete protein copy number used here breaks down anyway.

The Eq. (32) will be needed for derivation of the coefficient of variation of protein concentration, under the assumption that the burst size probability density function has the form (31), compatible with the exponential probability density function but not limited to it.

### Cell volume growth

In accordance with the experimental findings^34,35^ we assume that cell volume *V* grows exponentially:

33 $$\begin{aligned} V(\tau ) = V_0 e^{\lambda \tau }, \end{aligned}$$

where $V_0=V(0)$ is the volume of a newborn cell and $\lambda$ is cell volume growth rate. We ignore the stochastic spread of *T*, $\lambda$ and $V_0$^25,36^, hence the cell volume exactly doubles during the cell cycle ($V(T) = 2 V(0) = 2V_0$ and $\lambda = \ln (2)/T$). However, even if probability distributions of $V_0$, *V*(*T*) and $\lambda$ are not very broad for bacteria^36–38^, such an assumption is reasonable only as the first approximation.

### Effective protein copy number

In Ref.^1^, the authors have shown how the coefficient of variation of gene expression in *E. coli* scales with the mean protein level. The abundance of a fluorescent protein fusion produced from a given gene in a single cell was normalized by the volume of each individual cell to get the protein concentration. However, the final results have been presented in Ref.^1^ as *the effective protein copy number*, i.e., concentration multiplied by the average volume of cells in the population:

34 $$\begin{aligned} x^{\star } = {\tilde{x}} V_a = {\tilde{x}} \langle V(\tau ) \rangle _a, \end{aligned}$$

where $V_a = \langle V(\tau ) \rangle _a$ is the population-averaged cell volume.

From (34) it follows that the probability density function for the effective protein copy number $x^{\star }$ in the cell population is given by

35 $$\begin{aligned} p^{\star }_a (x^{\star }) = \frac{1}{V_a} {\tilde{p}}_a \left( \frac{x^{\star }}{V_a} \right) , \end{aligned}$$

where ${\tilde{p}}_a({\tilde{x}})$ is given by (29). The moments $\mu ^{\star }_{ra}$ of $p^{\star }_a (x^{\star })$ (for the effective protein copy number) depend on the moments ${\tilde{\mu }}_{ra}$ of ${\tilde{p}}_a({\tilde{x}})$ (for the protein concentration) in the following manner:

36 $$\begin{aligned} \mu ^{\star }_{ra} = \int _{0}^{\infty } (x^{\star })^{r} p^{\star }_a (x^{\star })d x^{\star } = V_a^{r} {\tilde{\mu }}_{ra}. \end{aligned}$$

In consequence, the protein concentration noise ${\tilde{c}}_{va}^2 = {\tilde{\kappa }}_{2a}/{\tilde{\mu }}_{1a}^2$ is not affected by the change of variables ${\tilde{x}} \rightarrow x^{\star }$:

37 $$\begin{aligned} {\tilde{c}}_{va}^2 = {\tilde{\kappa }}_{2a}/{\tilde{\mu }}_{1a}^2 = \kappa ^{\star }_{2a}/(\mu ^{\star }_{1a})^2 \equiv (c^{\star }_{va})^2. \end{aligned}$$

### Cell cycle dependent transcription rate

To show the dependence of protein noise on the timing of protein production, we consider transcription rate which is nonzero only during a fraction of the cell cycle:

38 $$\begin{aligned} k(\tau ) = k_{t} \ne 0; \,\,\,\,\alpha T< \tau \le \beta T, \,\,\,\, 0 \le \alpha < \beta \le 1, \end{aligned}$$

and zero otherwise. Note that $k(\tau )$ can be arbitrary. In fact, the abrupt change in protein production as given by (38) is not very realistic, but it allows us to study the influence of protein production variability during the cell cycle on the protein noise in an idealized and somewhat extreme case. For $k(\tau )$ (38), the quantity ${I}_r$ defined by Eq. (15) reads

39 $$\begin{aligned} {I}_r = k_{t} m_r (\beta - \alpha ) T = m_r (\beta - \alpha ) \Omega . \end{aligned}$$

The parameter $\Omega = k_{t} T$ defined in the above equation is the mean number of protein bursts per cell cycle if $\alpha = 0$ and $\beta = 1$; in a general case, the mean number of protein bursts per cell cycle is equal to $(\beta - \alpha )\Omega$ but in what follows for simplicity we still refer to $\Omega$ as the ‘mean number of bursts per cycle’.

If $k(\tau )$ is given by (38) then the mean effective protein copy number in the proliferating cell population, $\mu ^{\star }_{a1}$ (36), reads

40 $$\begin{aligned} \mu _{1a}^{\star }= & {} 2 \ln (2) (\beta -\alpha ) {A}_1 b \Omega , \,\,\,\, \Omega \equiv k_{t} T, \end{aligned}$$

where

41 $$\begin{aligned} {A}_1= & {} A_1 (\alpha , \beta ) = \frac{2^{-2 \alpha }-2^{-2 \beta }}{2 \ln (2) (\beta -\alpha )}+\frac{1}{2}. \end{aligned}$$

It is also convenient to define the following auxiliary functions, which will be used in the next section:

42 $$\begin{aligned} {A}_2= & {} A_2 (\alpha , \beta ) =\frac{2^{-3 \alpha }-2^{-3 \beta }}{3 \ln (2) (\beta -\alpha )}-\frac{1}{8}, \nonumber \\ {A}_3= & {} A_3 (\alpha , \beta ) = \frac{4 \left( 2^{-3 \alpha }-2^{-3 \beta }\right) }{27 \ln ^2(2) (\beta -\alpha )^2}+\frac{4 \left( 2^{-3 \alpha }-2^{1-3 \beta }\right) }{9 \ln (2) (\beta -\alpha )}+\frac{1}{3}. \end{aligned}$$

## Results

Using the model of stochastic gene expression in dividing cells described in the previous section, we calculate the coefficient of variation of protein concentration ${\tilde{c}}_{va}^2 = (c^{\star }_{va})^2$, which measures the protein noise (Eq. 37). We want to see how it depends on the mean protein abundance $\mu _{1a}^{\star }$.

In order to compare the model predictions with the experimental data of Ref.^1^, it is convenient to consider the two extreme cases: Frequency modulation (FM) and amplitude modulation (AM). In FM, the mean size *b* of translational bursts is constant in Eq. (40) so that the mean protein level $\mu _{1a}^{\star }$ can be changed only by changing the mean burst frequency $k_{t}$, or more generally by changing the mean number of bursts per cycle $\Omega = k_{t} T$. The corresponding expression for the protein noise will be given by $\Delta _F(\mu _{1a}^{\star }, b)$ in Eq. (43) below. In contrast, for AM, the mean burst size *b* varies and the mean number of bursts per cycle $\Omega$ is constant. The expression for the protein noise in that case will be given by $\Delta _A(\mu _{1a}^{\star }, \Omega )$ in Eq. (46) below.

### Frequency modulation

Using (28) we obtain the protein concentration noise ${\tilde{\kappa }}_{2a}/{\tilde{\mu }}^2_{1a} \equiv {\tilde{c}}^2_{va} = \Delta _F(\mu _{1a}^{\star }, b)$ (37) expressed as a function of the effective mean protein copy number $\mu _{1a}^{\star } = V_a {\tilde{\mu }}_{1a}= 2 \ln (2) V_0 {\tilde{\mu }}_{1a}$ (34) and the mean burst size $b=m_1$:

43 $$\begin{aligned} \Delta _F(\mu _{1a}^{\star }, b)= & {} \frac{7\ln (2)C_2 b}{18} \frac{ \left[ \chi _F(\mu _{1a}^{\star }, b) + 1 \right] }{ {A}_1\mu _{1a}^{\star }} + \frac{7}{12}\frac{\chi _F(\mu _{1a}^{\star }, b)}{ {A}_1^2} + \frac{4\ln (2)C_2 b}{3} \frac{ {A}_2}{ {A}_1 \mu _{1a}^{\star }} + \frac{ {A}_3}{ {A}_1^2} -1, \end{aligned}$$

where

44 $$\begin{aligned} \chi _F(\mu _{1a}^{\star }, b)\equiv h_1\left( {M}_2 \right) = \frac{4}{2 C_2 b + 3[\ln (2) {A}_1]^{-1}\mu _{1a}^{\star }}. \end{aligned}$$

Above, $A_1$, $A_2$ and $A_3$ are defined in Eqs. (41) and (42), $h_1( {M}_2)$ is given by (14) whereas ${M}_2$ is given by (32) with

45 $$\begin{aligned} \mu _1(T) = [\ln (2) {A}_1 ]^{-1} \mu _{1a}^{\star }. \end{aligned}$$

The relationship (45) between $\mu _{1a}^{\star }$ and $\mu _1(T)$ follows from Eqs. (10), (15), (16) and (40); note that now we have ${I}_1 = b (\beta - \alpha ) \Omega$.

### Amplitude modulation

Using Eq. (40), we rewrite the protein concentration noise $\Delta _F(\mu _{1a}^{\star }, b)$ (43) as a function of the effective mean protein copy number $\mu _{1a}^{\star }$ and $\Omega \equiv k_{t} T$ being the product of the mean translational burst frequency $k_{t}$ and cell cycle length *T*, where $(\beta - \alpha )\Omega$ is the mean number of bursts per cycle:

46 $$\begin{aligned} \Delta _A(\mu _{1a}^{\star }, \Omega )= & {} \frac{7 C_2 }{36} \frac{ \left[ \chi _A(\mu _{1a}^{\star }, \Omega ) + 1 \right] }{ {A}^2_1 (\beta - \alpha )\Omega } + \frac{7}{12}\frac{\chi _A(\mu _{1a}^{\star }, \Omega )}{ {A}_1^2} + \frac{2 C_2 }{3} \frac{ {A}_2}{ {A}^2_1 (\beta - \alpha )\Omega } + \frac{ {A}_3}{ {A}_1^2} -1. \end{aligned}$$

In the above equation, $\chi _A(\mu _{1a}^{\star }, \Omega ) = h_1( {M}_2) = \chi _F(\mu _{1a}^{\star }, b(\mu _{1a}^{\star }, \Omega ))$ (44) with $b(\mu _{1a}^{\star }, \Omega )$ given by (40). Note that the only dependence of $\Delta _A(\mu _{1a}^{\star }, \Omega )$ on $\mu _{1a}^{\star }$ comes from $\chi _A(\mu _{1a}^{\star }, b)$.

### Protein noise as dependent on the mean number of bursts per cell cycle and mean burst size

**Figure 1:**
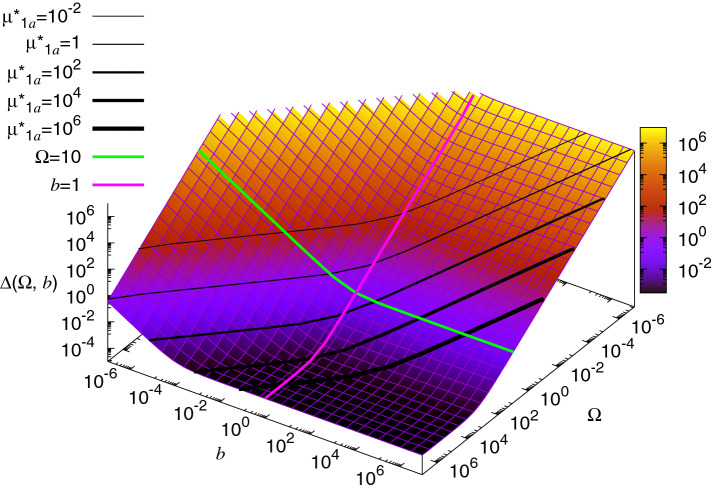
Protein noise $\Delta (\Omega , b)$ (47) as a function of the mean number of bursts per cell cycle $\Omega$ and mean burst size *b*. Black lines denote the levels of a constant mean protein abundance $\mu _{1a}^{\star }$, given by Eq. (40). $\mu _{1a}^{\star }$ may be varied by moving across these levels along some path defined as a dependence between *b* and $\Omega$. For example, the magenta line $b=const$ (here, $b=1$) denotes the increase in gene expression levels by increasing the mean burst frequency only (FM). Conversely, the green line $\Omega =const$ (here, $\Omega =10$) denotes the increase in gene expression levels by increasing the mean burst size only (AM).

The coefficient of variation of the protein concentration may be also written as explicitly dependent on both the mean number of bursts per cell cycle $\Omega$ and the mean burst size *b* but not on the effective mean protein copy number $\mu _{1a}^{\star }$ (Fig. 1):

47 $$\begin{aligned} \Delta (\Omega , b) = \frac{7}{18 (\beta - \alpha ) {A}^2_1 \Omega b} + \frac{C_2 }{36} \frac{(24 A_2 + 7)}{(\beta - \alpha ) {A}^2_1 \Omega } + \frac{ {A}_3}{ {A}_1^2} -1. \end{aligned}$$

Black lines in Fig. 1 mark the levels of a constant mean protein abundance (mean effective protein copy number) $\mu _{1a}^{\star }$, given by Eq. (40). $\mu _{1a}^{\star }$ may be varied by moving across these levels along some path which needs to be found experimentally as a dependence between the mean burst size *b* and mean burst frequency $\Omega$. The two simplest paths, $b=const$ (FM) and $\Omega =const$ (AM) have been proposed in the subsections above.

### Deterministic protein production

In order to quantify the contributions to protein noise it is desired to compare the predictions of the present model with the predictions of a similar model in which protein production is deterministic. If the protein production is not treated as stochastic but it is described by the deterministic source with intensity $\sigma (t) \ge 0$, then, instead of Eq. (1), we have

48 $$\begin{aligned} \partial _t p(x,t) = -\partial _x \left[ \sigma (t) p(x,t)\right] . \end{aligned}$$

Assuming $p(0,t)=0$, the Laplace transform of this equation reads $\partial _t {G}(s,t) = - \sigma (t) s G(s,t)$ and can be obtained from Eq. (2) by replacing $k(t)[{\hat{\nu }}(s, t) - 1]$ with $- \sigma (t) s$. (After the change of the time variable from *t* to $\tau$, this equation reads $\partial _{\tau } {G}(s|\tau ) = - \sigma (\tau ) s G(s|\tau )$, cf. (6) and (7).) Thus, all predictions of the model with deterministic protein production can be obtained from its stochastic counterpart by putting $\sigma (\tau ) = k(\tau )m_1(\tau )$ and $m_r = 0$ for $r > 1$, hence ${J}_2(\tau )= \,{I}_2=0$ in (28) or $C_2 = 0$ in (43) and (44). Note that now we have only a single function $\sigma (\tau )$ describing protein production instead of the two independent functions, $k(\tau )$ and ${\nu }(u | \tau )$, for the stochastic case.

Due to the condition $p(0,t)=0$, the model with deterministic protein production is not sufficient to describe the system of interest if the protein abundance is low.

### Comparison with experimental results

In this subsection, we compare the values of the coefficient of variation of protein concentration predicted by our model with the experimental data of Ref.^1^, to see under what conditions our model reproduces the measured scaling relation of protein noise vs. mean protein abundance.

**Figure 2:**
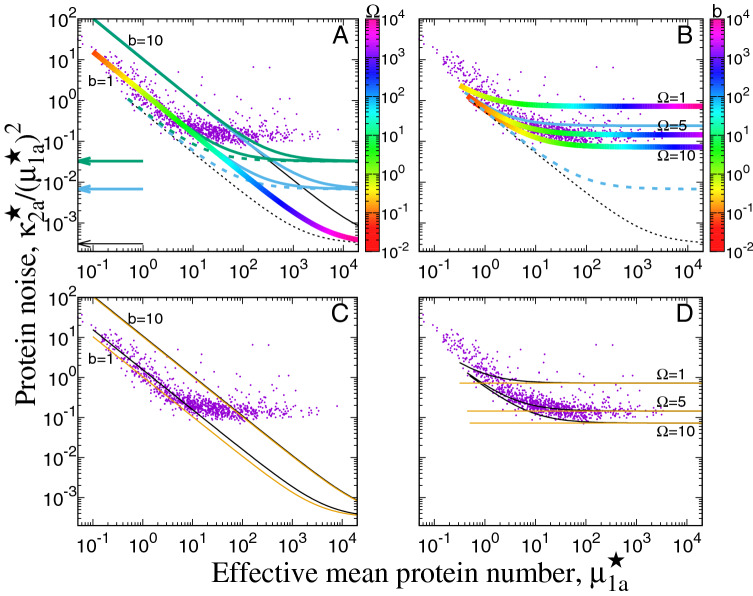
Protein noise vs. effective mean protein copy number against the experimental data of Ref.^1^ (dots). (**A**) Mean burst size $b = const$ (FM): $\Delta _F(\mu _{1a}^{\star }, b)$ (43). Solid lines: stochastic protein production ($C_2 =2$). Rainbow (color scale denotes the mean burst frequency values $\Omega$) and black: $\alpha = 0$, $\beta =1$ ($\epsilon =1$), blue: $\alpha = 0.2$, $\beta =0.8$ ($\epsilon =0.6$), green: $\alpha = 0.45$, $\beta =0.55$ ($\epsilon =0.1$). Dashed lines: deterministic protein production ($C_2 =0$) for $\alpha = 0$, $\beta =1$ ($\epsilon =1$). Arrows: Theoretical noise floor levels. (**B**) Mean burst number per cell cycle $\Omega = const$ (AM): $\Delta _A(\mu _{1a}^{\star }, \Omega )$ (46) with stochastic protein production ($C_2 =2$). Rainbow (color scale denotes the mean burst size *b*) and black: $\alpha = 0$, $\beta =1$ ($\epsilon =1$), blue: $\alpha = 0.2$, $\beta =0.8$ ($\epsilon =0.6$) for $\Omega =5$. Dashed lines: deterministic protein production ($C_2 =0$) for $\alpha = 0$, $\beta =1$ ($\epsilon =1$). (**C**) $\Delta _F(\mu _{1a}^{\star }, b)$ (43) with random protein partitioning (black) and half-by-half partitioning (yellow; close to black for $b=10$). (**D**) $\Delta _A(\mu _{1a}^{\star }, \Omega )$ (46) with random protein partitioning (black) and half-by-half partitioning (yellow). $C_2 =2$, $\alpha = 0$, $\beta =1$ in (**C**,**D**).

Both the extreme cases of the coefficient of variation in our model, $\Delta _F(\mu _{1a}^{\star }, b)$ for FM (43) and $\Delta _A(\mu _{1a}^{\star }, \Omega )$ for AM (46), have the ’boomerang’ shape, in a qualitative agreement with the data^1^, see Fig. 2. However, neither $\Delta _F(\mu _{1a}^{\star }, b)$ (43) with $b=const$ nor $\Delta _A(\mu _{1a}^{\star }, \Omega )$ (46) with $\Omega =const$ should be used for fitting to experimental data; these two functions are just cross-sections of $\Delta (\Omega , b )$ (47) along a fixed value of *b* (or $\Omega$), where the value of the non-fixed parameter, $\Omega$ (or *b*), is expressed by $\mu _{1a}^{\star }$ according to the constraint (40). For unambiguous fitting, one would need to additionally introduce an experimentally-based dependence of *b* or $\Omega$ on $\mu _{1a}^{\star }$, i.e., to define the cross-section path through $\Delta (\Omega , b )$ (Fig. 1), as a function of $\Omega$ and *b*. For this reason, we say that the agreement of the model with the data of Ref.^1^ is semi-quantitative because one can always define a constraint $b=b(\mu _{1a}^{\star })$ or $\Omega =\Omega (\mu _{1a}^{\star })$ such that the resulting cross-section path will fit the data. One of such constraints may be $\Omega =const$, as discussed below. In general, it seems reasonable that for small protein abundances (small $\mu _{1a}^{\star }$), when *b* cannot be too small, $\mu _{1a}^{\star }$ changes mainly due to varying $\Omega$ (FM). In Fig. 1, that would correspond to the increase in the mean protein abundance $\mu _{1a}^{\star }$ by moving down the coefficient of variation plot along the magenta line, $b=const$. We can also expect that, for highly expressed genes (large $\mu _{1a}^{\star }$), the values of $\Omega$ saturate (AM) due to some physical upper bound for $\Omega$. That would correspond to the transition from the magenta line $b=const$ to the green line $\Omega =const$ in Fig. 1 in order to further increase the mean protein abundance $\mu _{1a}^{\star }$. In Ref.^1^, Fig. 5A therein, the experimental data were effectively divided into such two regimes. However, clear distinction between the FM and AM regimes when comparing our model to the data is possible only if the cell-age dependence of the protein production is identical for all genes, which may be unrealistic: $k(\tau )$ and $\nu (u|\tau )$ should be ascribed individually for each gene.

And therefore, the apparent good agreement of the theoretical curves in Fig. 2B with the data of Ref.^1^ should be treated with caution. In the naive interpretation, the curves show that translational bursts from most genes have approximately the same mean number of several bursts per cell cycle and the gene expression levels vary only due to the variation of mean burst sizes (AM). However, as discussed above, this picture is too simple: Firstly, the most interesting curved part of the noise vs. mean plot in Fig. 2B falls, at least partly, for the values of $b < 1$ and such small mean burst sizes seem to be unphysical. Secondly, $k(\tau )$ is here assumed constant for each gene, which may not be true.

In the FM regime, the noise floor level ${F}(\alpha , \beta )$ is given by

49 $$\begin{aligned} {F}(\alpha , \beta )\equiv & {} \lim _{\mu _{1a}^{\star } \rightarrow \infty } \Delta _F(\mu _{1a}^{\star }, b) = \frac{ {A}_3(\alpha , \beta )}{ {A}_1(\alpha , \beta )^2}-1. \end{aligned}$$

${F}(\alpha , \beta )= {F}(\alpha , \alpha +\epsilon )$ depends strongly on $\epsilon = \beta -\alpha$ (Fig. 2A). But if there exists an upper bound for $\Omega$, in particular in the AM regime, where $\Omega = const$, then there is an additional contribution to noise floor given by the 2nd term of Eq. (47) and coming from 1st and 3rd terms in Eqs. (43) or (46), which may substantially enhance the noise floor level. This contribution, proportional to $C_2$, is due to the burst size distribution $\nu (u)$ (note that the squared coefficient of variation of $\nu (u)$ is equal $C_2 - 1$, and thus $C_2$ tells about the burst size distribution’s width). Comparing the case of stochastic ($C_2 > 0$) and deterministic ($C_2 =0$) protein production, we find the contribution of the the burst size distribution’s width to the total protein noise. The noise floor level is an increasing function of $C_2$ in the AM regime (Eq. 46). In the FM regime, the difference between stochastic and deterministic protein production is pronounced only for low and intermediate protein abundances but the noise floor does not depend on $C_2$ nor on *b* (Fig. 2A, Eq. (43)).

The noise floor levels in the AM regime, where they depend most strongly on $\Omega$, give an interesting information about the lowest possible protein production rates $k_t$ for highly expressed genes: The noise floor level of $\Delta _A(\mu _{1a}^{\star }, \Omega )$ for $\Omega =1$ lies well above most of the data (Fig. 2B), and therefore the burst frequency of highly expressed genes should be at least several bursts per cell cycle (depending on cell cycle length *T*). This is equivalent to the AM regime in^1^, Fig. 5A therein. However, that bound should be even higher if the transcription is cell-cycle dependent, $k(\tau )\ne const$ (Fig. 2B, blue line; we plotted only an example line for $\Omega =5$ and protein production during 0.6 of the cell cycle not to obscure the plot; the noise floor levels for $\Omega =1$ and $\Omega =10$ increase by a similar proportion), or if other extrinsic noise contributions (not included in our model) are present.

The noise floor level may be set by limiting the mean number of protein bursts per cell cycle $\Omega$: At that limit, any further increase in gene expression is obtained by increasing the burst size *b* (Fig. 2B). If, however, $\Omega$ were unlimited, then the noise floor given by (49) would fall well below the level observed in the experiment^1^, even for the extremely short period of gene expression, $\epsilon =0.1$ of the cell cycle (green line in Fig. 2A). Thus, in the FM regime, transcription during only a part of the cell cycle does not seem to realistically increase the noise floor up to the experimentally measured level (Fig. 2A). Note that the contribution from that effect is additive and the vertical scale for the coefficient of variation in the plots in Fig. 2 is logarithmic. The additive increase due to the limitation of gene expression to a part of the cell cycle is thus better visible for low noise but it becomes small for the experimentally measured noise levels. We can see this in the AM regime, where the noise floor is defined by a constant mean number of bursts per cell cycle $\Omega$ so that it can match the levels observed in experiment: The additional limitation of gene expression to $\epsilon =0.6$ of the cell cycle only slightly increases the noise floor level (Fig. 2B, blue line vs. rainbow line for $\Omega =5$). In our model, the minimal noise floor level is $\sim 3 \times 10^{-4}$ (Fig. 1A, FM): For $\alpha =0$, $\beta =1$ (protein production is constant during the entire cell cycle) we obtain the minimum of ${F}(\alpha , \beta )$ (49); ${F}(0,1)= \frac{4}{3} W(\ln 2)-1 \approx 0.00031$ where $W(\zeta ) = (\zeta ^2 + \zeta + \frac{7}{18})/(\zeta + \frac{3}{4})^2$. The corresponding *copy number* noise floor is equal to $1 - 2 (\ln 2)^2 \approx 0.03901$^15^. The fact that protein concentration noise is one or two orders of magnitude smaller than the corresponding protein copy number noise was also pointed out in Ref.^11^.

Random protein partitioning at cell division is the cause of the ’boomerang’ shape of the noise vs. mean plot in the AM regime. For the deterministic ’half-by-half’ partitioning with $\eta (q)=\delta (q-1/2)$ and ${M}_2 =1/4$, the plot in the AM regime is flat (Fig. 2D). In the FM regime, the plot has the ’boomerang’ shape even for the half-by-half partitioning, and the contribution of random partitioning to noise is small (Fig. 2C). In both AM and FM regimes, the noise floor level is not affected by the random partitioning. This is because ${M}_2(\mu _{1a}^{\star }) \rightarrow 1/4$ and thus $\chi _A(\mu _{1a}^{\star }, \Omega ) \rightarrow h_1(1/4) = 0$, $\chi _F(\mu _{1a}^{\star }, b) \rightarrow h_1(1/4) = 0$ when $\mu _{1a}^{\star } \rightarrow \infty$.

## Discussion

We have proposed a model of gene expression in a population of dividing cells which reproduces in a semi-quantitative manner the experimental data of Ref.^1^. In particular, our model predicts the existence of the noise floor, i.e., the absolute lower bound for protein noise. Within our model, there are three factors contributing to the noise floor: (i) cell volume and mean protein number may increase asynchronously, which leads to variation of mean protein concentration during cell cycle, (ii) transcription may take place during a fraction of cell cycle and (iii) a physical limitation may be imposed on the mean number of bursts per cell cycle. Although (ii) contributes to (i), we will discuss it separately.

Both cell volume growth and mean protein copy number growth are purely periodic and thus deterministic in our model, so is the mean protein concentration calculated with respect of the sub-population of cells of the same age. Consequently, the lack of synchronicity between time evolution of cell volume and mean protein copy number is also of purely deterministic character. This is evident if we note that an identical dependence of mean protein concentration, and thus the same contribution to noise floor, appears in the corresponding model with protein production being deterministic instead of stochastic. For that reason, the term ’noise’ may be slightly misleading in the case of (i). This is in analogy to the following situation: One can calculate the variance of a purely deterministic periodic, e.g., sinusoidal signal but the non-zero variance does not mean that the signal has any random component. The degree of randomness of such a signal can be measured by calculating its time correlation, if time-dependent data are available. Without the knowledge of time correlation, just looking at the squared coefficient of variation vs. mean plot of gene expression, one may see an apparent ’noise floor’ being the effect of an extrinsic periodic deterministic signal. More realistically, the effect of such a signal may occur as a contribution to the actual noise floor^11^.

However, in order to obtain the protein noise floor for the whole cell population in our model, we have to calculate the variance of the mean protein concentration with respect to the population age structure (probability distribution of cell age or generation time). For that reason, the stochastic character of (i) is related to the stochastic character of the population age structure. And therefore, (i) is a consequence of both the fact that not all cells are of the same age and that the mean protein concentration ${\tilde{\mu }}_1(\tau )$ varies with cell age $\tau$: The oscillations of the mean protein concentration in consecutive cell cycles occur when protein production does not keep up with or exceeds the cell volume growth. This is already possible for a constant transcription rate but when transcription is limited to a part of the cell cycle (ii), the noise floor level may increase even by 2 orders of magnitude (Fig. 2A).

Protein noise can be plotted as a function of mean protein abundance, $\mu _{1a}^{\star }$, after defining how the mean burst size *b* or the mean number of bursts per cycle $(\beta -\alpha )\Omega$ depends on $\mu _{1a}^{\star }$, with the extreme cases being the frequency modulation (FM, Fig. 2A,C) and amplitude modulation (AM, Fig. 2B,D).

The curved shape of the noise vs. mean plot for low protein abundances (tending to $\sim 1/\mu _{1a}^{\star }$, Poissonian limit) in the FM regime is due to the burst-like protein production and it occurs even for the deterministic and equal protein partitioning at cell division; random partitioning contributes weakly to the noise for realistic *b* values. For AM, the Poissonian limit is due to the random protein partitioning between daughter cells at cell division and it disappears when partitioning is deterministic and equal. For AM, the noise floor level depends on $\Omega$ (mean number of bursts per cycle for the case of $(\beta -\alpha ) = 1$, i.e., for the constant protein production taking place during the entire cell cycle) but it is also finite for $\Omega =\infty$ (deterministic case). Since the experimentally observed noise floor level is $\sim 10^{-1}$, the contribution to it coming solely from the age structure (i) and cell-cycle dependent gene expression (ii) seems to be very small compared to the contributions of the limitation on $\Omega$ (iii) (AM, Fig. 2A) or to the contributions of other possible sources of extrinsic noise not included in the model (e.g., generation time variability^28,29,36^ or cell growth rate variability^36^).

Protein noise is often decomposed into extrinsic and intrinsic contributions^39,40^. What is the character of each of the three noise sources (i–iii) considered here?

Within the present approach, cell volume growth and population age structure depend neither on the protein copy number nor on the kinetic parameters describing gene expression. Hence, the cell-cycle dependent variation of mean protein concentration due to asynchronous increases in cell volume and in mean protein number (i) are an extrinsic contribution to protein noise.

Now consider the effect of transcription during only a part of cell cycle in each cell generation (ii). Gene regulation, which leads to cell-cycle dependent gene expression, is extrinsic with respect to the gene of interest and deterministic in our model. But protein noise, which occurs when gene expression is enabled, is intrinsic. These notions are to be understood is in analogy those used in the classical works which disentangle extrinsic and intrinsic contributions to gene expression noise by means of the two-reporter assay^40^, where the regulator noise is considered extrinsic.

However, note that there are no correlations assumed *a priori* between different genes within our model, although such correlations might be present in a cell when a group of cell-cycle-dependent genes is expressed during the same part of cell cycle because of a common cell-cycle-dependent regulator. Such correlations may also occur due to the competition for polymerases (ribosomes) between different genes (transcripts). But in our model we treat each gene (each data point in Fig. 2) as independent, and possibly independently regulated by cell-cycle-dependent factors: For each gene on the plot, the cell-age-dependent transcription rate and the cell-age-dependent burst size distribution may be different. Thus, if we plot a theoretical curve corresponding to gene expression during a fraction of cell cycle against the experimental data (Fig. 2), it does not mean that all data points falling on the curve are the genes expressed during that fraction of cell cycle. It is possible that many theoretical curves can be drawn across the same data point, as corresponding to gene expression during different fractions of cell cycle (or as corresponding to gene expression with different values of other parameters). This shows that our model cannot be used for fitting the data without additional information that would remove ambiguity. The necessary information includes: (a) Experimental dependence linking mean expression level with both mean size and mean frequency of protein bursts. (b) Dependence of transcription rate on cell-cycle age. (c) Dependence of the burst size distribution on cell-cycle age.

Note also that protein production limited to a fraction of the cell cycle (ii) enhances asynchrony between cell volume and mean protein copy number and therefore it contributes to (i).

Finally, consider any limitation imposed on the mean number of protein bursts per cell cycle (iii). If such limitation is due to the limitations imposed on transcription rate, this should be treated as an intrinsic contribution because it depends solely on the parameters describing the gene of interest. Obviously, the contribution (iii) does not appear in the corresponding model where gene expression is a deterministic process. In the deterministic approach, we have a single protein production rate parameter $\sigma$ describing gene expression instead of two independent parameters (transcription rate $k_t$ and mean burst size *b*) for the stochastic approach. (For simplicity we refer to a situation when gene expression is time-independent). Without these two parameters it is impossible to fit the stochastic model to experimental data, even semi-quantitatively.

If the number of protein bursts per cycle is small, the contribution to noise (iii) is much larger than both (i) and (ii), but if bursts are small and frequent then (iii) either alone or with (i) and (ii) is too small to explain the observed noise floor level.

In summary, our model includes some of the factors contributing to protein noise in gene expression and to the noise floor in particular. Although the model is sufficient for obtaining the functional dependences between the mean protein abundance and noise which apparently fit the experimental data of Ref.^1^, it does not take into account some important contributions to protein noise like stochastic spread of cell volume at birth, cycle length or growth rate of individual cells. As these noise sources are of extrinsic character, the protein noise floor is likely to be of mostly extrinsic origin, too. Still, we show that the sources of protein noise included in our model suffice to obtain the noise floor, and we quantify their contributions to protein noise.

## References

[1] Taniguchi Y (2010). Quantifying e. coli proteome and transcriptome with single-molecule sensitivity in single cells. Science.

[2] Silander OK (2012). A genome-wide analysis of promoter-mediated phenotypic noise in escherichia coli. PLoS Genetics.

[3] Nordholt N, van Heerden J, Kort R, Bruggeman FJ (2017). Effects of growth rate and promoter activity on single-cell protein expression. Scientific Reports.

[4] Newman JR (2006). Single-cell proteomic analysis of s. cerevisiae reveals the architecture of biological noise. Nature.

[5] Bar-Even A (2006). Noise in protein expression scales with natural protein abundance. Nature Genetics.

[6] Keren L (2015). Noise in gene expression is coupled to growth rate. Genome Research.

[7] Volfson D (2006). Origins of extrinsic variability in eukaryotic gene expression. Nature.

[8] Salman H (2012). Universal protein fluctuations in populations of microorganisms. Physical Review Letters.

[9] Hausser J, Mayo A, Keren L, Alon U (2019). Central dogma rates and the trade-off between precision and economy in gene expression. Nature Communications.

[10] Dar RD, Razooky BS, Weinberger LS, Cox CD, Simpson ML (2015). The low noise limit in gene expression. PloS One.

[11] Marathe R, Bierbaum V, Gomez D, Klumpp S (2012). Deterministic and stochastic descriptions of gene expression dynamics. Journal of Statistical Physics.

[12] Cole JA, Luthey-Schulten Z (2017). Careful accounting of extrinsic noise in protein expression reveals correlations among its sources. Physical Review E.

[13] Berg OG (1978). A model for the statistical fluctuations of protein numbers in a microbial population. Journal of Theoretical Biology.

[14] Huh D, Paulsson J (2011). Random partitioning of molecules at cell division. Proceedings of the National Academy of Sciences.

[15] Schwabe A, Bruggeman FJ (2014). Contributions of cell growth and biochemical reactions to nongenetic variability of cells. Biophysical Journal.

[16] Bruggeman FJ, Schouten J, de Groot DH, Planqué R (2019). Cell fate determination by lamarckian molecule-inheritance and chance. bioRxiv.

[17] Walker N, Nghe P, Tans SJ (2016). Generation and filtering of gene expression noise by the bacterial cell cycle. BMC Biology.

[18] Soltani M, Singh A (2016). Effects of cell-cycle-dependent expression on random fluctuations in protein levels. Royal Society open science.

[19] Friedman N, Cai L, Xie XS (2006). Linking stochastic dynamics to population distribution: an analytical framework of gene expression. Physical Review Letters.

[20] Feng H, Hensel Z, Xiao J, Wang J (2012). Analytical calculation of protein production distributions in models of clustered protein expression. Physical Review E.

[21] Jedrak J, Ochab-Marcinek A (2016). Time-dependent solutions for a stochastic model of gene expression with molecule production in the form of a compound poisson process. Physical Review E.

[22] Brenner N, Shokef Y (2007). Nonequilibrium statistical mechanics of dividing cell populations. Physical Review Letters.

[23] Friedlander T, Brenner N (2008). Cellular properties and population asymptotics in the population balance equation. Physical Review Letters.

[24] Jędrak J, Kwiatkowski M, Ochab-Marcinek A (2019). Exactly solvable model of gene expression in a proliferating bacterial cell population with stochastic protein bursts and protein partitioning. Physical Review E.

[25] Bokes P, Singh A, Bortolussi L, Sanguinetti G (2019). Cell volume distributions in exponentially growing populations. Computational Methods in Systems Biology.

[26] Jafarpour F (2019). Cell size regulation induces sustained oscillations in the population growth rate. Physical Review Letters.

[27] Powell EO (1956). Growth rate and generation time of bacteria, with special reference to continuous culture. Journal of General Microbiology.

[28] Thomas P (2017). Making sense of snapshot data: ergodic principle for clonal cell populations. Journal of The Royal Society Interface.

[29] Thomas P (2019). Intrinsic and extrinsic noise of gene expression in lineage trees. Scientific reports.

[30] Quedeville V, Morchain J, Villedieu P, Fox RO (2019). A critical analysis of powell’s results on the interdivision time distribution. Sci. Rep..

[31] Cai L, Friedman N, Xie XS (2006). Stochastic protein expression in individual cells at the single molecule level. Nature.

[32] Yu J, Xiao J, Ren X, Lao K, Xie XS (2006). Probing gene expression in live cells, one protein molecule at a time. Science.

[33] Choi PJ, Cai L, Frieda K, Xie XS (2008). A stochastic single-molecule event triggers phenotype switching of a bacterial cell. Science.

[34] Lin J, Amir A (2018). Homeostasis of protein and mrna concentrations in growing cells. Nature communications.

[35] Susman L (2018). Individuality and slow dynamics in bacterial growth homeostasis. Proceedings of the National Academy of Sciences.

[36] Lin J, Amir A (2017). The effects of stochasticity at the single-cell level and cell size control on the population growth. Cell systems.

[37] Osella M, Nugent E, Lagomarsino MC (2014). Concerted control of escherichia coli cell division. Proceedings of the National Academy of Sciences.

[38] Ho P-Y, Lin J, Amir A (2018). Modeling cell size regulation: From single-cell-level statistics to molecular mechanisms and population-level effects. Annual review of biophysics.

[39] Swain PS, Elowitz MB, Siggia ED (2002). Intrinsic and extrinsic contributions to stochasticity in gene expression. Proceedings of the National Academy of Sciences.

[40] Elowitz MB, Levine AJ, Siggia ED, Swain PS (2002). Stochastic Gene Expression in a Single Cell. Science.

